# Latest Advancements of Natural Products in Combating Ovarian Cancer

**DOI:** 10.7150/jca.118209

**Published:** 2025-07-28

**Authors:** Yangyuxiao Lu, Hong Huang, Yuling Chen, Keren He, Mengqi Fang, Ye Xu, Yuan Zhou, Fangfang Tao

**Affiliations:** 1 The First Affiliated Hospital of Zhejiang Chinese Medical University (Zhejiang Provincial Hospital of Chinese Medicine), Hangzhou, China.; 2 School of Basic Medical Sciences, Zhejiang Chinese Medical University, Hangzhou, China.

**Keywords:** ovarian cancer, natural products, therapeutic strategy, molecular mechanisms, traditional Chinese medicine.

## Abstract

Ovarian cancer (OC), the seventh most common cancer in women globally, is one of the most prevalent and lethal gynecological cancers. With a high degree of malignancy and a dismal prognosis, late-stage patients frequently experience relapses and metastases. Associated with this is the high drug resistance of OC, which is a common challenge in cancer treatment. Natural products have garnered increasing attention in tumor treatment due to their relatively high safety and stable sources. Previous studies have demonstrated that various active ingredients in natural products can impact the progression of OC through diverse molecular mechanisms. These mechanisms include inducing cell cycle arrest, regulating reactive oxygen species (ROS), modulating autophagy, promoting cell apoptosis, inhibiting proliferation, invasion, and migration of OC cells, and regulating the tumor immune microenvironment (TIME). This article comprehensively elucidates the mechanisms by which natural products combat ovarian cancer and highlights both the emerging opportunities and existing challenges in their development as effective therapeutic agents.

## The Genetic and Epigenetic Characteristics of OC

Ovarian cancer (OC), recognized as one of the most aggressive gynecological malignancies, ranks seventh globally in incidence among female cancers 1. While its incidence remains relatively stable, persistently high mortality rates pose significant public health challenges 2. Updated 2024 data from the American Cancer Society reveals that the 5-year relative survival rate for OC patients remains below 50%, underscoring the disease's grave clinical prognosis 3. According to the National Comprehensive Cancer Network (NCCN) clinical practice guidelines, surgery combined with platinum-based chemotherapy continues to serve as the first-line standard treatment regimen 4. In recent years, immune checkpoint inhibitors (ICIs) have emerged as the most promising therapeutic innovation, demonstrating substantial clinical benefits both as monotherapy and in combination with conventional modalities including radiotherapy and chemotherapy 5. However, treatment for OC still faces certain limitations. Advanced-stage disease frequently manifests as extra-ovarian disseminated metastases characterized by extensive peritoneal carcinomatosis and high recurrence rates 2. Furthermore, the development of multidrug resistance in tumor cells constitutes a critical pathological mechanism underlying therapeutic failure and unfavorable prognoses 6.

OC exhibits intricate molecular pathogenesis characterized by heterogeneous genetic and epigenetic alterations, involving dysregulated signaling pathways, somatic gene mutations, and aberrant epigenetic modifications. Current studies have been identified recurrent genetic abnormalities, notably including functional inactivation of tumor suppressor genes (e.g., BRCA1/2 and TP53 mutations) 7, 8, and oncogenic amplification events (e.g., RAD21 gene amplification) 9, which collectively drive tumor progression. Epigenetic dysregulation encompasses DNA methylation abnormalities, histone post-translational modifications, and non-coding RNA (ncRNA) disturbances. These epigenetic mechanisms orchestrate the transcriptional activity of oncogenesis-related genes, thereby modulating critical carcinogenic processes including neoplastic initiation, metastatic dissemination, and therapeutic resistance 10. Particularly noteworthy is the overexpression of miR-205 in OC tissues, which demonstrates significant associations with enhanced metastatic potential and serves as an independent prognostic biomarker for adverse outcomes 11.

## Source and Phytochemical Inventory of Anti-ovarian Cancer Herbs

In recent years, the application value of traditional Chinese medicine (TCM) formulations based on holistic principles has become increasingly prominent in the comprehensive treatment system for OC. Representative classical prescriptions, such as Guizhi Fuling Formula, Lichong Shengsui Decoction, and Fufang Banmao Capsule, demonstrate synergistic antitumor effects through the scientific combination of medicinal components like Ginseng, Astragalus, and Cinnamon. Modern pharmacological research has revealed that these formulations exhibit significant advantages in inhibiting tumor proliferation, inducing apoptosis, and reversing drug resistance through multi-target regulatory mechanisms 12.

Taking Astragalus as an example, its core active component, quercetin, has been demonstrated in studies on anti-ovarian cancer mechanisms to exert its effects by inducing endoplasmic reticulum (ER) stress, apoptosis, and autophagy through the p- signal transducer and activator of transcription 3 (STAT3)/Bcl-2 axis 13. Notably, when quercetin is combined with the PARP inhibitor (PARPi) olaparib, a synergistic enhancement effect is observed, significantly improving the anti-proliferative efficacy against BRCA wild-type OC cells. This provides a crucial theoretical foundation for integrated traditional Chinese and Western medicine treatment strategies 14.

In addressing chemotherapy resistance, the active component of ginger, 6-Shogaol, demonstrates unique advantages. It overcomes drug resistance in OC cells by inducing ER stress and cell death 15. Ginsenosides exert their effects through multimodal mechanisms: Rg3 inhibits the migration of SKOV3 cells 16, while 20(S)-Rg3 further suppresses SKOV3 tumor metastasis by inducing autophagy 17. Additionally, ginsenoside 20(S)-Rg3 blocks hypoxia-induced epithelial-mesenchymal transition (EMT) in OC cells by downregulating hypoxia-inducible factor-1α (HIF-1α) expression 18.

The aforementioned research findings have established a solid theoretical foundation for the clinical application of TCM formulations in the treatment of OC. The structurally diverse family of natural compounds holds significant promise as a source of novel candidate drugs for OC therapy. Among these, plant-derived natural products, owing to their unique structural complexity and chemical diversity, demonstrate irreplaceable core value in the development of innovative therapeutic agents 19.

## Bioactive Components in Anti-OVC Herbs

A comprehensive overview of the chemical structures and biological actions of the natural products discussed in this study is presented in Table 1. Analysis of these natural products reveals that the majority possess polyphenolic structures, including compounds such as tanshinone, quercetin, and ellagic acid. These compounds have demonstrated efficacy in improving outcomes in OC through various mechanisms, including the modulation of cell proliferation, autophagy, apoptosis, and several other signaling pathways. These findings are consistent with structure-activity relationship analyses, which highlight the positive correlation between the number of phenolic hydroxyl groups and antioxidant activity, as well as the enhanced transmembrane permeability conferred by lactone ring structures. Furthermore, the chemical classification of compounds such as terpenoids, flavonoids, and alkaloids further supports their potential anticancer properties. Additionally, these results underscore the impact of variations in extraction processes on the proportional composition of active components 20.

This article explores the potential role of natural products in the treatment of OC. Based on their mechanisms of action, the natural products with reported anti-ovarian cancer activity are systematically classified, summarized, and discussed. These natural products primarily function in cytotoxicity, inducing cell cycle arrest, regulating reactive oxygen species (ROS), modulating autophagy, promoting cell apoptosis, inhibiting the proliferation, invasion, and migration of OC cells, inhibiting angiogenesis, and regulating the tumor immune microenvironment (TIME).

**Figure 1:**
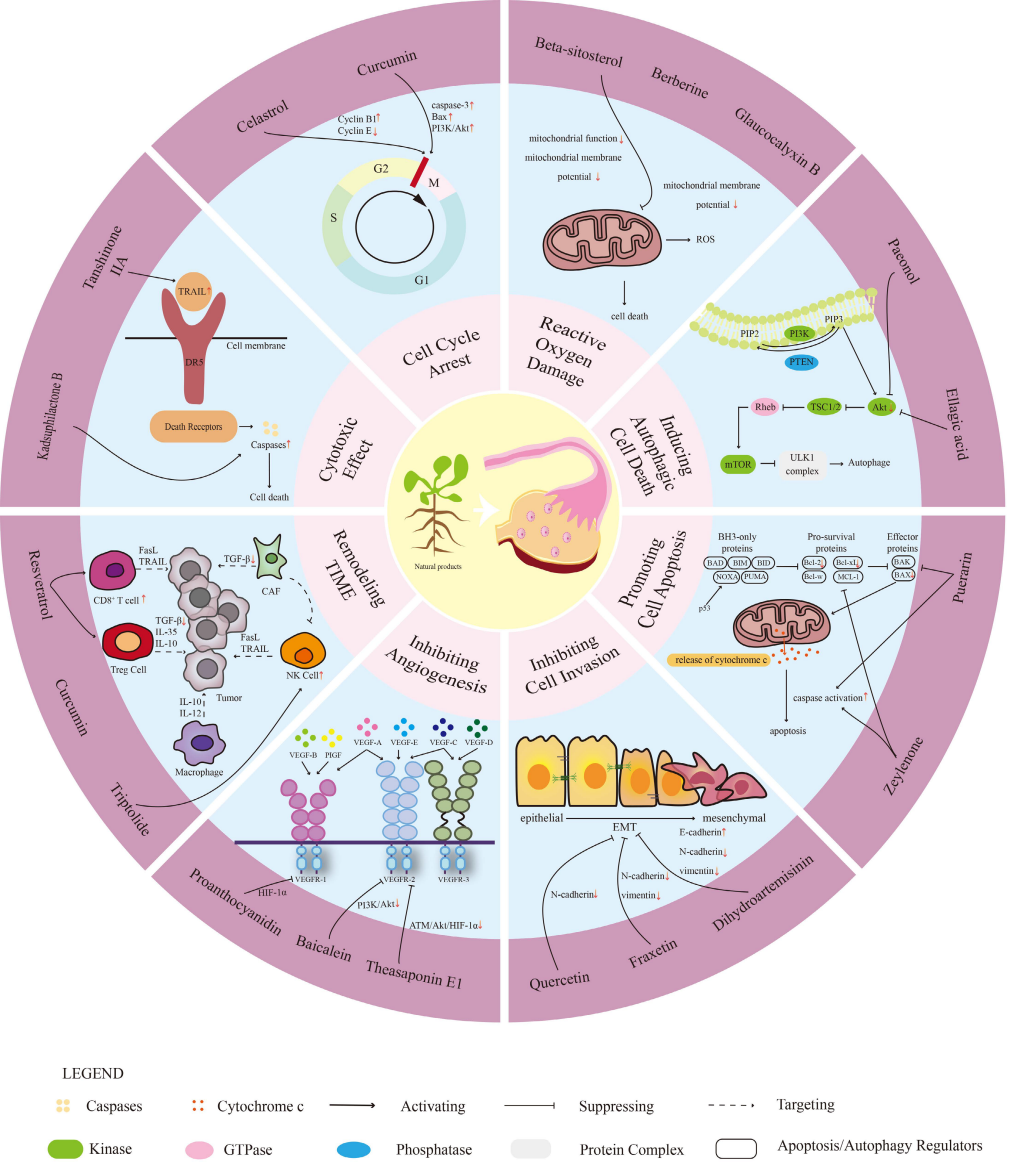
The mechanism of natural products in ovarian cancer (OC). This diagram illustrates the multifaceted mechanisms through which natural products may influence OC biology. The concentric hierarchical progression highlights the key roles of natural products: (1) exhibiting cytotoxicity, (2) inducing cell cycle arrest, (3) promoting reactive oxygen species (ROS)-mediated damage, (4) triggering autophagy, (5) activating apoptotic pathways, (6) reducing proliferation and migration, (7) inhibiting angiogenesis, and (8) modulating the tumor immune microenvironment. These mechanisms collectively act to slow tumor progression and may improve patient outcomes while preventing recurrence. Through these pathways, natural products emerge as a promising therapeutic approach for managing OC.

In the future, natural drugs are expected to become important tools in the treatment of OC, providing more effective and safer treatment options. The molecular mechanisms are shown in Figure 1.

## Search Strategy

The literature search was conducted in PubMed using a combination of Medical Subject Headings (MeSH) terms and free-text keywords related to OC, natural product interventions, and mechanistic pathways (e.g., autophagy, apoptosis, PI3K/Akt/mTOR signaling). Inclusion criteria encompassed *in vitro*, *in vivo*, and clinical trial studies published within the last decade that explicitly investigated the mechanisms of natural products in OC regulation. Exclusion criteria removed incomplete data, irrelevant studies, and non-peer-reviewed publications. To ensure comprehensive coverage, citation tracking of highly cited references and backward/forward citation searches of high-impact publications were performed. After deduplication, titles and abstracts were screened to exclude non-relevant studies, followed by full-text assessment of remaining articles against predefined criteria. A standardized data extraction form was designed to systematically capture key information, including experimental models, compound concentrations, and molecular targets. Two independent researchers executed the search and screening processes to ensure rigor, with discrepancies resolved through discussion or third-party arbitration.

## Biological Effects of Natural Products

### Cytotoxic Effects

#### Cytotoxic Drug-Based Therapeutic Strategies for OC

The cytotoxic suppression of cancer cells primarily achieves synergistic antitumor effects by targeting critical biological processes such as DNA replication, cell division, metabolism, or signaling. DNA double-strand breaks (DSBs), the most lethal form of DNA damage 21, can be induced by chemotherapy or radiotherapy, triggering poly (ADP-ribose) polymerase (PARP)-dependent base excision repair (BER). When PARP inhibitors (PARPi) block BER and form "PARP-DNA trapping complexes," unrepaired single-strand breaks (SSBs) are converted into DSBs during replication, leading to selective elimination of homologous recombination repair (HRR)-deficient tumor cells via synthetic lethality 22. Notably, PARPi not only directly kill cancer cells through DNA damage but also activate the natural immune response, driving a Th1-polarized immune response (e.g., IFN-γ secretion), promoting dendritic cell maturation and T-cell infiltration, and synergizing with immune checkpoint inhibitors 23.

Additionally, TNF-related apoptosis-inducing ligand (TRAIL) activates caspase cascades by binding death receptors (DR4/DR5), with selective induction of apoptosis 24. Based on these mechanisms, dual-targeting strategies for OC can be designed as follows: 1) Combined Induction of DNA Damage and Repair Inhibition—using natural products to induce DSBs while inhibiting DNA damage response (DDR) key proteins (e.g., PARP), triggering synthetic lethality through irreversible damage accumulation; 2) Death Receptor Signaling Remodeling—epigenetically upregulating DR4/DR5 expression while suppressing anti-apoptotic factors (e.g., c-FLIP/XIAP) to enhance TRAIL-mediated apoptotic sensitivity, thereby overcoming therapeutic resistance in OC.

#### Cytotoxic Natural Products with anti-Ovarian Cancer Activity

Recent pharmacological studies delineate both direct and indirect mechanisms underlying natural compounds' anti-ovarian cancer effects. A representative example is kadsuphilactone B, a Schisandra chinensis-derived nortriterpenoid exhibiting moderate cytotoxicity against A2780 cells (IC_50_ <25 μM). Mechanistic investigations revealed concentration-dependent activation of both intrinsic and extrinsic apoptotic pathways, evidenced by sequential cleavage of caspase-9/-8/-3 cascade effectors and PARP. Pharmacological inhibition of caspases significantly attenuated the pro-apoptotic effects, confirming caspase-dependent apoptosis. Western blot analysis further identified concomitant modulation of B-cell lymphoma 2 (Bcl-2) family proteins and phosphorylation alterations in mitogen-activated protein kinase (MAPK) signaling pathways, suggesting multimodal regulation of programmed cell death 25.

Synergistic cytotoxic effects were observed in TOV-21G endometrial cancer models through combined therapy. While monotherapy with 100 ng/mL TRAIL induced 21.1% ± 3.6% cell death, coadministration with 2 μM Tanshinone IIA (TIIA) potentiated cytotoxicity to 88.5% (Δ +5.6% vs theoretical additive effect). This chemosensitization correlated with enhanced proteolytic processing of apoptotic mediators: immunoblotting demonstrated marked elevation of cleaved caspase-9/-7/-3 and PARP fragments in combination groups compared to single-agent treatments. These data establish that TIIA directly targets the TRAIL-induced apoptosis pathway by caspase cascade amplification, rather than indirect metabolic or immune modulation, providing rationale for combination regimens in apoptosis-resistant malignancies 26.

### Cell Cycle Arrest

#### Inducing Cell Cycle Arrest as a Therapeutic Strategy for OC

The progression of OC is closely linked to dysregulation of cell cycle control, which is tightly governed by three critical checkpoints: G1/S phase, intra-S phase, and G2/M phase checkpoints 27. Upon DNA damage, checkpoint kinase systems—including the Rad3-related serine/threonine kinase (ATR)-checkpoint kinase 1 (CHK1) signaling axis and WEE1 G2 checkpoint kinase (WEE1)—are activated to induce cell cycle arrest, providing a time window for DNA repair and maintaining genomic stability 28. In OC pathogenesis, despite its multifactorial complexity, disruption of the cell cycle regulatory network has been identified as a core pathogenic mechanism. This dysregulation disrupts the dynamic equilibrium between aberrant proliferation and programmed cell death, ultimately driving malignant proliferation and metastatic progression in ovarian tumors 29.

Targeted therapies centered on cell cycle regulation have become pivotal in OC treatment. CDK4/6 inhibitors, suppress tumor proliferation by blocking the G1/S phase transition through competitive binding to the ATP domain of CDK4/6, thereby inhibiting phosphorylation of the retinoblastoma protein (Rb) and inducing irreversible G1 arrest. This arrest not only halts cell cycle progression but also amplifies antitumor effects by driving metabolic reprogramming and transcriptomic remodeling 30. In parallel, WEE1 inhibitors release the G2/M phase arrest by preventing Tyr15 phosphorylation of CDK1/2, forcing replication-stressed tumor cells into abnormal division 31. Clinically, combining CDK4/6 inhibitors with PARP inhibitors has shown synergistic efficacy, as this dual strategy simultaneously disrupts cell cycle checkpoints and DNA repair pathways—particularly effective in tumors with HRR deficiencies 32. By leveraging synthetic lethality and precision biomarker-guided approaches, these therapeutic advancements offer new avenues to improve clinical outcomes for OC patients 33.

#### Anti-Ovarian Cancer Natural Products Inducing Cell Cycle Arrest

Emerging evidence highlights the cell cycle-modulating potential of phytochemicals in OC therapeutics. Curcumin, a bioactive polyphenol isolated from Curcuma longa rhizomes (Zingiberaceae family), demonstrates multimodal cell cycle disruption in SKOV-3 ovarian adenocarcinoma. Pharmacological studies revealed that 30 μM curcumin triggered a time-dependent accumulation of apoptotic sub-G1-phase cells (no significant change at 6 hours, but marked increases at 24 and 48 hours). At 6 hours, S- and G2/M-phase populations increased, followed by reductions at 24 and 48 hours. Notably, curcumin treatment significantly decreased the percentage of G1/G0-phase cells across all timepoints. However, prolonged exposure (24-48 hours) to 30 μM curcumin resulted in a global reduction of cells in G1/G0, S, and G2/M phases, suggesting progressive cell cycle arrest and apoptotic commitment 34. Mechanistically, this biphasic cell cycle arrest coordinates with pro-apoptotic signaling amplification through PI3K/Akt axis modulation, evidenced by upregulation of caspase-3 activity and elevation of Bcl-2-associated X protein (Bax), effectively impairing cancer cell self-renewal capacity 35.

Furthermore, curcumin directly induces cancer cell apoptosis by triggering mitochondrial accumulation, oxidative stress, and elevated DNA damage 36. Additionally, it selectively suppresses the hyperactivated Wnt/β-catenin signaling pathway in OC, synergistically enhancing chemotherapeutic drug sensitivity 37.

The triterpenoid celastrol, derived from Tripterygium wilfordii radix, emerges as another potent cell cycle modulator. The compound demonstrates multifaceted antiproliferative effects, inducing cell cycle arrest through distinct molecular targets while simultaneously provoking direct cell cycle perturbation via complementary pathways. Flow cytometric analysis of A2780/SKOV-3 co-cultures exposed to 0.3, 1, and 3 μM celastrol revealed an increase in the sub-G1 population accompanied by proportional reduction of G0/G1 phase cells, demonstrating significant cell cycle progression disruption. Molecular profiling revealed cyclin network reprogramming upregulation of p27 and Cyclin B1 contrasted with reduction in Cyclin E expression. This confirms celastrol's ability to induce dual-phase cell cycle arrest through cyclin-dependent kinase (CDK) inhibitor (CDKi) activation and G2/M checkpoint potentiation 38.

### Inducing Reactive Oxygen Damage

#### Inducing Reactive Oxygen Damage as a Therapeutic Strategy for OC

In cells, oxidative stress arises from an imbalance between ROS generation and antioxidant defenses. ROS—including superoxide radicals, hydrogen peroxide, and hydroxyl radicals—induce genomic instability through DNA alkylation, phosphodiester bond cleavage, and strand breaks, leading to mutations, chromosomal translocations, and impaired repair mechanisms. These lesions drive carcinogenesis by disrupting cell cycle checkpoints, suppressing apoptosis, and promoting oncogenic activation (e.g., KRAS, PI3K) while silencing tumor suppressors (e.g., TP53, BRCA1), collectively fostering a pro-tumorigenic microenvironment 39-42.

Ferroptosis is a form of regulated cell death characterized by iron-dependent lipid peroxidation, with its molecular basis involving dysregulation of glutathione (GSH) metabolism and collapse of the membrane phospholipid repair system. Mechanistic studies have shown that GSH peroxidase 4 (GPX4) maintains membrane stability by reducing phospholipid hydroperoxides (PLOOH), while GPX4 inhibitors can induce PLOOH accumulation and trigger oxidative disintegration of the plasma membrane 43. Research has found that combined treatment with GPX4 inhibitors and PARPi exhibits synergistic antitumor efficacy in BRCA1-deficient OC cells, patient-derived organoids (PDOs), and xenografts 44, providing an important direction for drug development and clinical treatment of OC.

#### Anti-Ovarian Cancer Natural Products Inducing Reactive Oxygen Damage

Berberine, a quaternary protoberberine alkaloid isolated from Berberis species, exhibits potent cytotoxic effects in OC cells. At a concentration of 30 μM, berberine induces cell death in nearly 90% of SKOV-3 cells. Furthermore, when combined with gamma-ray irradiation, berberine enhances oxidative stress, as evidenced by elevated ROS levels and reduced GSH content. This dual targeting of cancer cell viability (direct cytotoxicity) and metabolic adaptation (indirect microenvironment modulation) establishes berberine as a promising radiosensitizer for OC therapy 45.

Glaucocalyxin B, a bioactive diterpenoid isolated from the aerial parts of Rabdosia japonica, demonstrates direct anticancer properties mediated through ROS-dependent pathways in OC cells. Studies demonstrate that glaucocalyxin B significantly elevates intracellular ROS levels in both cisplatin-sensitive (A2780) and cisplatin-resistant (A2780/DDP) cell lines. This ROS accumulation induces DNA damage and suppresses cancer cell proliferation via activation of the c-Jun N-terminal kinase (JNK) signaling pathway, which triggers mitochondrial apoptosis. These findings highlight glaucocalyxin B as a promising therapeutic candidate, particularly for overcoming cisplatin resistance in OC 46.

Beta-sitosterol exerts anti-ovarian cancer effects through both direct mitochondrial targeting and indirect stress-inducing mechanisms. This phytosterol not only triggers loss of mitochondrial membrane potential (MMP) but also activates key pro-apoptotic factors including Bax, Bcl-2 homologous antagonist/killer (BAK), caspase-3, caspase-9, and cytochrome C release. These coordinated events directly execute mitochondrial-mediated apoptosis in OC cells. Furthermore, Notably, beta-sitosterol also initiates indirect tumor-suppressive effects by disturbing cellular homeostasis. It significantly elevates intracellular ROS levels while disrupting calcium homeostasis, collectively generating oxidative stress that exacerbates mitochondrial damage and ultimately leads to programmed cell death in OC cells 47.

Artemisinin and its derivatives, natural compounds derived from Artemisia annua with established antimalarial and anticancer properties 48, demonstrate significant potential as ferroptosis inducers in OC therapy. These compounds exhibit concentration-dependent dual mechanisms: at lower concentrations, artemether induces ROS-independent G1 phase cell cycle arrest through mTOR signaling inhibition, while higher concentrations trigger ROS-mediated apoptosis and ferroptosis 49. The ferroptotic effects are primarily mediated through iron metabolism dysregulation, characterized by increased ferrous ion accumulation, GSH depletion, and elevated oxidative stress. As clinically viable ferroptosis-targeting agents, artemisinin compounds exert potent anti-tumor effects by promoting iron-dependent lipid peroxidation and subsequent cancer cell death 50. As a representative derivative of artemisinin compounds, preclinical studies have demonstrated that artesunate not only effectively inhibits the *in vitro* proliferation of OC cell lines and primary patient-derived cells but also significantly suppresses tumor growth in murine xenograft models by modulating the tumor microenvironment (TME)^51^. These findings systematically elucidate the molecular mechanisms through which artemisinin and its derivatives exert anti-ovarian cancer effects via spatiotemporal dynamic regulation of cell cycle progression, oxidative stress homeostasis, and ferroptosis signaling networks. This comprehensive evidence offers promising therapeutic potential for OC treatment either as monotherapy or in combination regimens.

### Inducing Autophagic Cell Death

#### Inducing Autophagic Cell Death as a Therapeutic Strategy for OC

In the pathogenesis and progression of OC, aberrant activation of the phosphatidylinositol 3-kinase/protein kinase B/mammalian target of rapamycin (PI3K/Akt/mTOR) signaling pathway is closely associated with genetic alterations. Gain-of-function mutations in the PIK3CA gene enhance PI3K lipid kinase activity, significantly elevating phosphorylation levels of Akt and Mek1/2, thereby promoting tumor cell survival and malignant transformation. Concurrently, copy number alterations resulting from PIK3CA gene amplification are strongly linked to hyperactivation of the PI3K pathway in tumor tissues. Oncogenic mutations in downstream effectors of the AKT family (AKT1/2/3) disrupt interactions between the pleckstrin homology domain (PH) and kinase domain (KD), leading to constitutive activation and driving mTOR complex 1 (mTORC1)-mediated anabolic reprogramming. As a critical negative regulator, inactivating mutations or epigenetic silencing of phosphatase and tensin homolog (PTEN) impair its phosphatase function, causing abnormal accumulation of phosphatidylinositol-3,4,5-trisphosphate (PIP3) and sustained activation of the Akt/mTOR signaling cascade, ultimately fostering tumor proliferation and metabolic adaptation. Notably, PTEN also modulates homologous recombination repair efficiency by regulating RAD51 expression. The mTORC1 complex suppresses autophagosome formation by phosphorylating Unc-51-like kinase 1 (ULK1) and the master lysosomal gene regulator transcription factor EB (TFEB), while simultaneously inhibiting lysosomal biogenesis to impair cellular clearance capacity. Under stress conditions, the p53 protein dynamically balances these processes through subcellular localization-dependent mechanisms, with nuclear translocation activating a pro-autophagy transcriptional network 52, 53.

Natural products hold promising potential to modulate tumor cell autophagy by targeting the PI3K/Akt/mTOR signaling pathway. Specifically, these compounds are expected to inhibit mTORC1 activity, thereby relieving its phosphorylation-mediated suppression of key autophagy factors. This dynamic regulation of lysosome-dependent degradation mechanisms helps maintain intracellular homeostasis. Notably, in malignant OC cells, such compounds may selectively inhibit hyperactivated autophagic pathways, impairing the clearance efficiency of damaged organelles and abnormal protein aggregates. Ultimately, this process activates apoptotic signaling to delay the progression of malignant phenotypes in OC.

#### Anti-Ovarian Cancer Natural Products Inducing Autophagic Cell Death

Paeonol, a natural compound extracted from Moutan Cortex, was found to significantly upregulate the expression of the autophagy marker Bax protein in treated A2780 and SKOV-3 OC cells, accompanied by the accumulation of autophagosomes and lysosomes, confirming the activation of intact autophagic flux. Paeonol relieves negative regulation on the autophagy initiation complex initiates by inhibiting the Akt/mTOR signaling pathway. Notably, moderate activation of paeonol-induced autophagy promotes stress adaptation in tumor cells, whereas prolonged intervention leads to autophagy-associated programmed cell death. Further validation demonstrated that combined application of autophagy inhibitors blocks the Akt/mTOR-dependent compensatory autophagy mechanism, significantly enhancing the anti-ovarian cancer efficacy of paeonol through synergistic amplification of apoptotic signaling 54.

Ellagic acid inhibited cell proliferation (IC_50_ = 36.6 μmol/L), migration, and invasion in a concentration-dependent manner, and significantly induced caspase-dependent apoptosis. It upregulated the expression of Beclin-1, ATG-5, and LC3-I/II while reducing p62 protein levels, indicating the complete activation of autophagic flux. Additionally, ellagic acid markedly increased the expression of the pro-apoptotic protein Bax and suppressed Bcl-2 levels, synergistically activating caspase-3/8 cleavage and modulating the balance of Bcl-2 family proteins. Further mechanistic analysis revealed that ellagic acid inhibited the PI3K/Akt/mTORC1 signaling axis, as evidenced by significant reductions in phosphorylated Akt and downstream targets of mTORC1, while activating the energy-sensing adenosine monophosphate-activated protein kinase (AMPK) pathway. Importantly, co-treatment with the autophagy inhibitor chloroquine partially abrogated the biological effects of ellagic acid, suggesting autophagy-dependence of its mechanism 55.

### Inhibiting Cell Proliferation and Promoting Cell Apoptosis

#### Targeting Cell Growth and Apoptosis in OC Therapy

The Bcl-2 protein family plays a central role in tumorigenesis by regulating the apoptosis pathway. Pro-apoptotic Bcl-2 homology 3 (BH3)-only proteins antagonize anti-apoptotic members such as Bcl-2, B-cell lymphoma-extra large (Bcl-xL), myeloid cell leukemia 1 (MCL-1), and Bcl-2-like protein 2 (Bcl-w) through high-affinity binding. This interaction activates Bax/BAK oligomerization, alters MMP, triggers cytochrome c release, and activates the caspase cascade, ultimately inducing apoptosis 56. This process is regulated through multiple layers by transcription factors such as E2F transcription factor 1 (E2F-1) and nuclear factor-kappa B (NF-κB). The Janus kinase (JAK)/signal transducer and activator of transcription (STAT) pathway enhances tumor cell survival signaling by promoting pro-survival members of the Bcl-2 protein family transcription. Additionally, post-translational modifications dynamically regulate protein stability 57.

In OC, natural products targeting apoptosis regulators, activating BAX/BAK to induce oligomerization, MMP, and cytochrome c release, thereby reactivating the caspase cascade and significantly increasing apoptosis rates in OC SKOV-3 cells. These natural products concurrently suppress the JAK/STAT3 pathway, offering synergistic intervention strategies to overcome chemotherapy resistance. Furthermore, combining such natural products with platinum-based chemotherapy agents enhances therapeutic potential through apoptosis signal reprogramming in OC treatment.

#### Anti-Ovarian Cancer Natural Products Inhibiting cell proliferation and promoting cell apoptosis

The polyketide compound Zeylenone, derived from Uvaria grandiflora, significantly activated the mitochondrial apoptosis pathway in OC cells after 24-hour treatment at concentrations of 2.5, 5, and 10 μg/mL. This effect was characterized by increased levels of the apoptosis-inducing factor (AIF) and suppression of Bcl-2 expression. Additionally, it initiates the extrinsic death receptor pathway via upregulating Fas/FasL expression, thereby triggering caspase cascade activation. Mechanistically, this dual pro-apoptotic effect is achieved through indirect regulation of the tumor microenvironment. Zeylenone significantly inhibits JAK/STAT survival signaling, and this upstream blockade subsequently disrupts downstream survival pathways 58.

*In vitro* experimental results demonstrated that puerarin exhibits growth inhibitory effects on OC cell lines SKOV-3 and Caov-4, with IC_50_ values of 157.0 μg/mL and 119.3 μg/mL, respectively. Notably, at a subtoxic concentration of 50 μg/mL, puerarin significantly induced apoptosis in SKOV-3 OC cells. Further analysis revealed that puerarin treatment markedly increased early and late apoptosis rates in both SKOV-3 cells and their cisplatin-resistant counterpart SKOV-3/DDP. Its direct mechanism of action is manifested as follows: puerarin reshaped the regulatory balance of the mitochondrial apoptosis pathway by downregulating anti-apoptotic proteins Bcl-2 and Bcl-xL, while reducing the expression of pro-apoptotic protein Bax. Morphological observations confirmed characteristic apoptotic changes, accompanied by cleavage of PARP protein, indicating activation of the caspase-dependent apoptosis pathway. Importantly, the mechanism of drug resistance reversal is related to the indirect regulation of the TME. Puerarin reversed cisplatin resistance in OC cells, a mechanism closely linked to inhibition of aberrant Wnt/β-catenin signaling pathway activation 59.

### Inhibiting Cell Migration and Invasion

#### Limiting Cell Migration and Invasion in OC Therapy

EMT, a core process driving tumor invasion and metastasis, induces transcriptional reprogramming via TGF-β/Smad and Wnt/β-catenin signaling pathways 60. This leads to reduced E-cadherin expression, elevated vimentin levels, and ultimately loss of cell polarity and enhanced stromal invasiveness 61, 62. Studies have demonstrated that using the TGF-β receptor kinase inhibitor Galunisertib^63^ or Wnt pathway inhibitors 60 can suppress EMT progression and reduce metastatic lesions. Clinical research aims to leverage natural products to restore epithelial phenotypes in OC cells by targeting mesenchymal markers and inhibiting EMT, thereby significantly lowering the risk of OC metastasis.

#### Anti-Ovarian Cancer Natural Products Inhibiting cell migration and invasion

Quercetin, a natural flavonoid compound, exerts direct anti-metastatic effects by targeting EMT core mechanisms in OC PA-1 cells. Specifically, it reverses the EMT phenotype through significant downregulation of the mesenchymal marker N-cadherin, a key driver of cellular motility and invasiveness 64.

The natural coumpound fraxetin, a coumarin derivative from Fraxinus rhynchophylla Hance, exerts direct anti-EMT effects in OC through primary targeting of the Toll-like receptor 4/STAT3 (TLR4/STAT3) signaling axis. This direct inhibition leads to significant downregulation of mesenchymal markers (N-cadherin, vimentin, c-Myc) and cell cycle regulators (Cyclin D1) in SKOV-3 and SW626 cells treated with 80 μM fraxetin for 12 hours. Notably, the anti-EMT effects of fraxetin could be partially reversed by the STAT3 activator Colivelin, confirming the critical role of STAT3 signaling in mediating its activity 65.

Dihydroartemisinin demonstrates various anti-metastatic mechanisms in OC through both direct molecular targeting and indirect microenvironmental modulation. Mechanistically, dihydroartemisinin directly suppresses EMT in A2780 and SKOV-3 cells by regulation of key EMT markers: upregulating epithelial signature protein E-cadherin while downregulating mesenchymal markers N-cadherin and vimentin. This EMT inhibition is synergistically reinforced through dihydroartemisinin's direct modulation of apoptotic machinery, specifically by reducing anti-apoptotic Bcl-2 expression, elevating pro-apoptotic Bax/Bcl-2 ratio, and activating executioner caspase-3 - constituting a cancer cell apoptosis pathway. Notably, dihydroartemisinin extends its therapeutic effects through indirect regulation of TME components. It significantly enhances expression of reversion-inducing cysteine-rich protein with Kazal motifs (RECK), a tumor suppressor that indirectly inhibits metastasis. These coordinated actions on both cancer cell-intrinsic pathways (direct EMT/apoptosis regulation) and extrinsic microenvironmental factors establish dihydroartemisinin as a multi-targeted therapeutic agent against OC progression 66.

### Inhibiting Angiogenesis

#### Inhibiting Angiogenesis as a Therapeutic Strategy for OC

Based on Folkman's tumor angiogenesis theory, the progression of OC relies on vascular endothelial growth factor (VEGF)-mediated pathological angiogenesis 67, with serum VEGF levels significantly elevated in OC patients compared to healthy controls 68. Current anti-angiogenic therapies inhibit endothelial cell proliferation and vascular permeability by targeting VEGF/VEGF receptor (VEGFR) binding 69. Emerging strategies are exploring the application of natural products to modulate the VEGF/VEGFR pathway, aiming to enhance the vascular normalization index and thereby improve therapeutic efficacy against OC.

#### Anti-Ovarian Cancer Natural Products Inhibiting angiogenesis

Theasaponin E1, a camellane-type saponin derived from Camellia seeds, exerts anti-angiogenic effects through modulation of the ATM/Akt/HIF-1α signaling axis. At the direct-action level, this compound specifically induces phosphorylation of ataxia-telangiectasia mutated (ATM) kinase (a key DNA damage response kinase) while upregulating PTEN expression. These dual mechanisms collectively suppress the Akt/mTOR signaling cascade, leading to reduced nuclear translocation of HIF-1α and inhibition of VEGF transcriptional activity. *In vivo* chorioallantoic membrane (CAM) assays demonstrated reduced neovascular density, with synergistic effects observed when combined with the Notch1 inhibitor DAPT or Akt inhibitors 70.

As a polyphenolic natural product, Myrica rubra leaf-derived proanthocyanidins demonstrate direct therapeutic mechanisms in OC intervention. The primary action involves targeting the AKT/mTOR/p70S6K/4E-BP1 signaling axis, which specifically downregulates hypoxia-inducible factor HIF-1α, thereby blocking VEGF transcriptional activation. Experimental data revealed that 10 μg/mL proanthocyanidins treatment significantly inhibited VEGF secretion in A2780/CP70 cells by 80.3%, accompanied by reduced *in vitro* angiogenesis capacity 71.

Baicalein, a natural flavonoid derived from Scutellaria baicalensis Georgi roots, exerts direct anti-tumor effects on OC by targeting the PI3K/Akt signaling axis. This primary mechanism leads to the direct suppression of angiogenesis through VEGF downregulation and inhibits metastatic progression by reducing Cyclin D1 and matrix metalloproteinase-2 (MMP2) expression. Furthermore, baicalein induces protective autophagy in OC cells by upregulating Beclin 1 and activating extracellular signal-regulated kinase (ERK)-mediated pathways, a dual-edged mechanism that initially promotes cancer cell survival but ultimately sensitizes cells to apoptosis under prolonged treatment 72. Notably, the compound may also indirectly modulate tumor progression, as evidenced by its significant tumor volume reduction following oral administration 73.

Notably, the anti-angiogenic mechanisms described above—particularly HIF-1α/VEGF suppression via PI3K/Akt/mTOR inhibition—have been substantiated by *in vivo* and early-phase clinical studies. For example, proanthocyanidins and baicalein, through these pathways, have demonstrated tumor growth inhibition in xenograft models. Such results underscore the feasibility of leveraging mechanistic insights to guide preclinical candidate selection and rational design of future clinical trials targeting angiogenesis in OC.

### Tumor Immune Microenvironment

The TIME in OC is characterized by a dynamic interplay of tumor cells, immune infiltrates, stromal components, and soluble mediators. Immune evasion within this ecosystem is a major barrier to durable therapeutic responses 74. Natural products offer promising immunomodulatory effects that can reprogram the TIME from an immunosuppressive to an immune-active state.

#### Modulation of adaptive immune cells

Several natural products have been shown to enhance cytotoxic T lymphocyte (CTL) activity while simultaneously suppressing regulatory T cell (Treg) function. For example, resveratrol promotes dendritic cell (DC) maturation (upregulating CD80/CD86) and CD8⁺ T cell activation by reducing lactate accumulation in the tumor milieu, which otherwise dampens T cell function. This dual metabolic and immunological remodeling leads to decreased TGF-β levels and increased IFN-γ expression. Such compounds restore antigen presentation and reinvigorate CTL-mediated anti-tumor immunity 75, 76.

#### Reprogramming tumor-associated macrophages (TAMs)

In OC, TAMs are often skewed toward the M2 phenotype, supporting tumor progression via immunosuppressive cytokine production (e.g., IL-10, TGF-β) and suppression of T cell activity 77. Natural compounds such as curcumin have demonstrated the ability to reprogram TAMs from the M2 to M1 phenotype, enhancing pro-inflammatory cytokine release (TNF-α, IL-12) and antigen presentation 78. This polarization shift is frequently mediated via suppression of STAT3 and activation of NF-κB pathways, ultimately supporting a more robust immune response 79.

#### Enhancement of natural killer (NK) cell cytotoxicity

Natural products may also reverse NK cell dysfunction induced by factors such as TGF-β within the TME. Triptolide has been shown to elevate systemic IL-2 and TNF-α levels, thereby restoring NK cell cytotoxic function. This boost innate immune surveillance and supports early tumor elimination 80.

#### Interference with immune checkpoint pathways

Some phytochemicals may indirectly inhibit immune checkpoint molecules such as PD-L1 via modulation of oncogenic pathways like PI3K/Akt or STAT3. Although direct evidence in OC is still emerging, these interactions present an opportunity for combining natural products with immune checkpoint inhibitors to synergistically remodel the immune landscape.

Together, these findings suggest that natural compounds exert multi-dimensional control over the TIME by restoring CTL activity, inhibiting Treg infiltration, reprogramming TAM polarization, and reviving NK cell function. This immunological rebalancing offers a promising avenue to overcome immune escape and enhance the efficacy of conventional and immunotherapeutic strategies in OC.

## The Molecular Mechanism of Natural Products

Emerging preclinical evidence highlights the multifaceted pharmacological mechanisms of natural products in OC therapeutics, particularly through their dual capacity as pathway modulators and chemo-sensitizing agents. Mechanistically, these bioactive compounds demonstrate oncogenic pathway activation potential via selective potentiation of STAT3 signaling and PI3K/Akt/mTOR axis regulation 81-83, while concurrently enhancing platinum-based chemotherapy through coordinated p53 modifying, thereby synergistically overcoming chemoresistance and amplifying cytotoxic efficacy 84. Through multi-target synergistic mechanisms, natural products exert therapeutic effects in suppressing tumor proliferation and inducing apoptosis by regulating pathway interactions, while concurrently remodeling the chemoresistant TME. This integrated pharmacological action ultimately enhances therapeutic response rates and optimizes clinical outcomes for OC patients.

**Figure 2:**
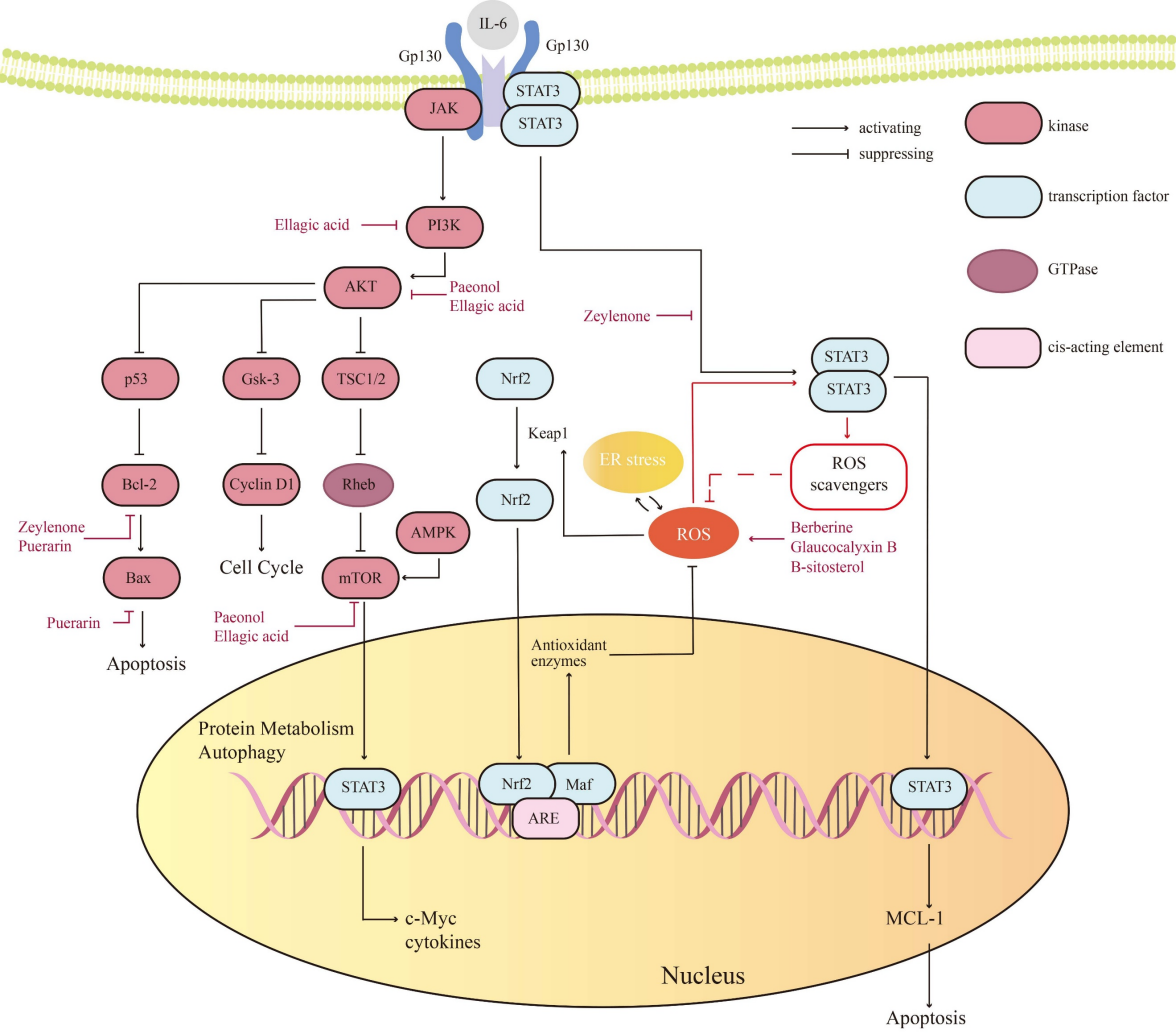
Key signaling pathways and proteins of natural products in ovarian cancer (OC).

The sophisticated regulation of cellular homeostasis relies on the Kelch-like ECH-associated protein 1-nuclear factor erythroid 2-related factor 2-Antioxidant Response Elements (Keap1-Nrf2-ARE) pathway governing redox equilibrium, mTOR-driven anabolic metabolism coupled with autophagy suppression, the Bcl-2 family-modulated apoptosis switch, and the JAK/STAT-mediated transcriptional regulatory network. Specifically, Keap1 dynamically regulates Nrf2 protein stability through ROS-sensing mechanisms to activate the antioxidant defense system 85, 86, while the mTOR pathway integrates growth factor and nutrient signals to sustain proliferative dominance via metabolic reprogramming. Concurrently, the Bcl-2 family coordinates mitochondrial membrane permeability (MOMP) regulation with JAK/STAT-mediated transcriptional activation of pro-survival factors to establish apoptotic resistance. These interconnected pathways collectively dictate tumor cell proliferation, stress adaptation capacity, and therapeutic resistance phenotypes, thereby delineating critical molecular interfaces for developing targeted combination therapies against OC (Figure 2).

## Bridging Mechanisms and Clinical Applications

To enhance translational relevance, we highlight several natural compounds with dual evidence from both mechanistic and clinical/preclinical studies. These compounds represent promising candidates for integration into evidence-based, multi-targeted OC treatment strategies.

## Natural Products in Clinical Applications of OC

Natural products with well-defined mechanisms are increasingly transitioning from preclinical research to clinical applications in ovarian cancer treatment. Notably, compounds such as epigallocatechin gallate (EGCG), resveratrol—previously shown to modulate apoptosis, and immune remodeling—have demonstrated promising outcomes in early clinical trials. Below, we summarize representative studies that bridge mechanistic understanding with translational and clinical validation in OC patients. While conventional chemotherapy regimens like paclitaxel-based combinations continue to demonstrate efficacy in trials such as ICON4/AGO-OVAR-2.2^87^ and GCGS-01^88^, showing improved progression-free survival (PFS) and overall survival (OS), emerging evidence highlights the complementary potential of natural product-based interventions. Notably, clinical studies have shown that EGCG-rich green tea consumption induced complete remission in advanced OC patients 89, while mistletoe extracts improved quality of life with minimal adverse effects 90, 91. Agaricus blazei mushroom extracts have exhibited immunomodulatory properties by enhancing NK cell activity and mitigating chemotherapy toxicity 92. Resveratrol has demonstrated particular promise through its multifaceted mechanisms - modulating glycolytic enzymes like PKM2 and GLUT1, suppressing TGF-β signaling, and enhancing cytotoxic T-cell responses - with preclinical studies confirming reduced tumor burden and early-phase clinical trials supporting its safety as an adjunct therapy 93. These findings collectively underscore how natural products may offer dual benefits in ovarian cancer management: potentiating conventional treatments while reducing their adverse effects, thereby presenting new opportunities for integrative therapeutic approaches.

## Conclusion and Perspectives

Natural products have demonstrated considerable anti-ovarian cancer potential through diverse mechanisms, yet face clinical translation challenges including poor bioavailability, limited tumor specificity, and potential toxicity 94. To address these limitations, researchers are developing structurally optimized derivatives through systematic structure-activity relationship studies - for instance, artemisinin derivatives exhibit enhanced pharmacokinetics and potent ferroptosis induction in OC cells 50 - while advanced delivery systems like liposomes and polymeric nanoparticles improve solubility, stability, and targeted delivery. Particularly promising are nanocarriers co-loaded with natural products and chemotherapeutics that show synergistic effects against drug-resistant tumors 94. However, critical challenges remain including variability in sourcing and processing methods, potential development of resistance during prolonged treatment, and most importantly the need for validation in large-scale clinical trials 95. Future research must prioritize comprehensive structure-activity relationship (SAR) studies to guide rational compound design, continued development of targeted delivery technologies, and rigorous clinical evaluation through well-designed trials to establish safety and efficacy. By integrating these chemical, pharmaceutical and clinical approaches, natural product derivatives may emerge as valuable components of ovarian cancer therapy with improved effectiveness and reduced toxicity compared to current treatments.

## Figures and Tables

**Table 1:** Natural plant components for anti-ovarian cancer effects.

Compound	Formula	Structure	Experiment	Cell types	IC_50_	Effect	Reference
Kadsuphilactone B	C_30_H_42_O_5_	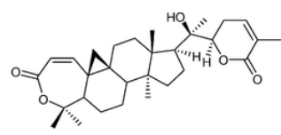	*In vitro*	A2780	Below 25 μM	Cytotoxic action	25
Tanshinone IIA	C_19_H_18_O_3_	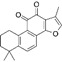	*In vitro*	TOV-21G, SKOV3, OVCAR3	N/A	Cytotoxic action	26
Curcumin	C_21_H_20_O_6_	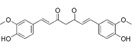	*In vitro*	SKOV3	24.8μM	Trigger cell cycle arrest, modulating the TIME, DNA damage	34-37, 96-98
Celastrol	C_29_H_38_O_4_	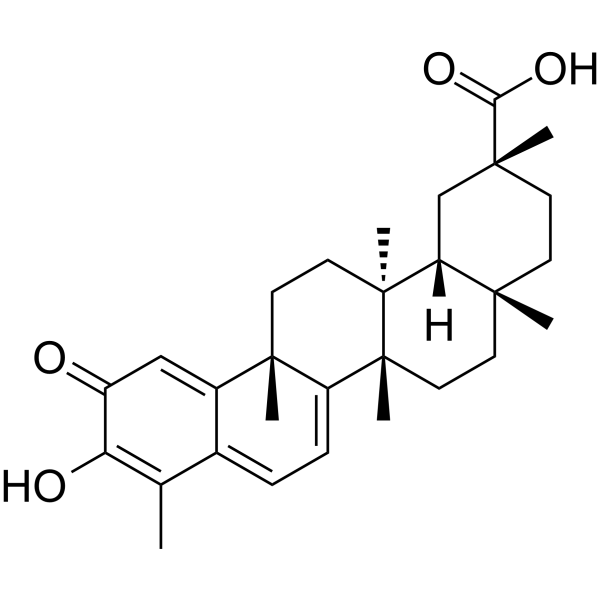	*In vitro* and *In vivo*	A2780	2.11μM	Trigger cell cycle arrest, modulating the TIME, inhibit proliferation, promote apoptosis and suppress cell migration and invasion	38
				SKOV3	2.29μM		
Berberine	C_20_H_18_NO_4_^+^	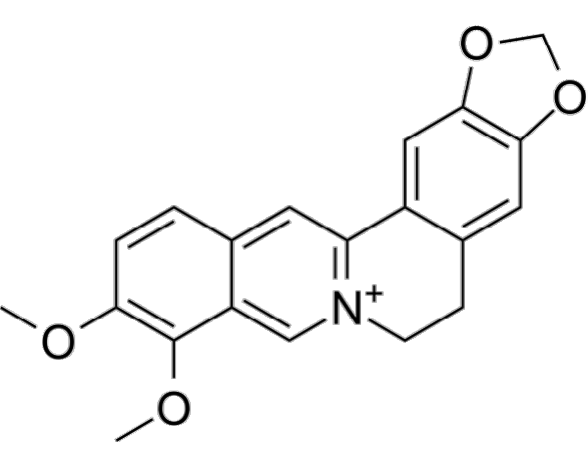	*In vitro*	SKOV3	N/A	ROS damage, promote DNA damage response	45
Glaucocalyxin B	C_22_H_30_O_5_	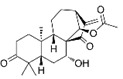	*In vitro*	A2780	N/A	ROS damage	46
Βeta-sitosterol	C_29_H_50_O	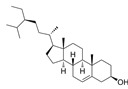	*In vitro*	ES2, OV90	N/A	ROS damage and promote apoptosis	47
Artemether	C_16_H_26_O_5_	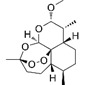	*In vitro* and *In vivo*	SKOV3	23.55±3.86μM	ROS damage, regulating apoptosis and ferroptosis	48-51
				HEY1	5.80±1.62μM		
				HEY2	7.34±0.56μM		
				OVCAR8	5.51±1.06μM		
				IGROV-1	8.82±1.18μM		
				OVCAR3	14.95±6.38μM		
				OV-90	31.89±4.15μM		
				TOV-21G	6.11±0.64μM		
Paeonol	C_9_H_10_O_3_	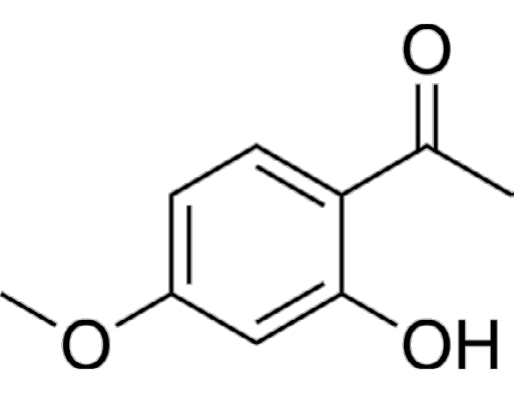	*In vitro* and *In vivo*	A2780, SKOV3	1.2mM	Autophagy	54
Ellagic acid	C_14_H_6_O_8_	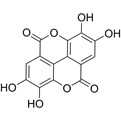	*In vitro*	SKOV3	36.6μM	Induce autophagy, inhibit proliferation and promote apoptosis, suppress cell migration and invasion	55
Puerarin	C_21_H_20_O_9_	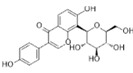	*In vitro* and *In vivo*	SKOV3	157.0μg/mL	Inhibit proliferation and promote apoptosis	59
				Caov-4	119.3μg/mL		
Quercetin	C_15_H_10_O_7_	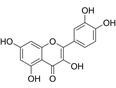	*In vitro*	PA-1	N/A	Suppress cell migration and invasion	64
Fraxetin	C_10_H_8_O_5_	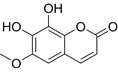	*In vitro*	SKOV3, SW626	N/A	Suppress cell migration and invasion	65
Dihydroartemisinin	C_15_H_24_O_5_	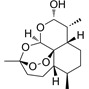	*In vitro*	OVCAR3, SKOV3, A2780, OV-56	80μmol/L	Suppress cell migration and invasion, inhibit proliferation	66
Proanthocyanidins	Compound	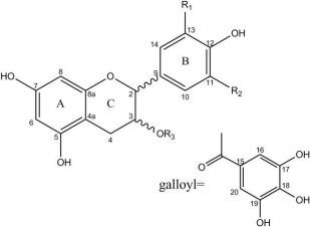	*In vitro*	A2780/CP70	N/A	Inhibit angiogenesis	71
Baicalein	C_15_H_10_O_5_	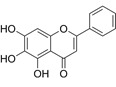	*In vitro* and *In vivo*	SKOV3	N/A	Inhibit angiogenesis and proliferation, autophagy	72, 73
Theasaponin E1	C_59_H_90_O_27_	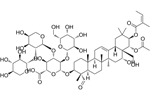	*In vitro*	OVCAR-3	3.5μM	Inhibit angiogenesis and proliferation	70
				A2780/CP70	2.8μM		
Triptolide	C_20_H_24_O_6_	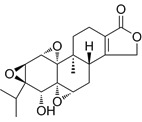	*In vitro* and *In vivo*	SKOV3/DDP	N/A	Suppress cell migration and invasion	80

## Abbreviations

AIF: apoptosis-inducing factor
Akt: protein kinase B
AMPK: adenosine monophosphate-activated protein kinase
ARE: antioxidant response elements
ATM: ataxia telangiectasia mutated
ATR: Rad3-related serine/threonine kinase
BAK: Bcl-2 homologous antagonist/killer
Bax: Bcl-2-associated X protein
Bcl-2: B-cell lymphoma 2
Bcl-w: Bcl-2-like protein 2
Bcl-xL: B-cell lymphoma extra-large
BER: base excision repair
BH3: Bcl-2 homology 3
CAM: chorioallantoic membrane
CDK: cyclin-dependent kinase
CDKi: CDK inhibitor
CHK1: checkpoint kinase 1
CTLs: cytotoxic T lymphocytes
DCs: dendritic cells
DDR: DNA damage response
DSBs: DNA double-strand breaks
E2F-1: E2F transcription factor 1
EGCG: epigallocatechin gallate
EMT: epithelial-mesenchymal transition
EOC: epithelial ovarian cancer
ER: endoplasmic reticulum
ERK: extracellular signal-regulated kinase
Fas: fatality-associated protein
FBXW7: F-box and WD-40 structural domain protein 7
GPX4: glutathione peroxidase 4
GSH: glutathione
Her2: human epidermal growth factor receptor 2
HRR: homologous recombination repair
HIF-1α: hypoxia inducible factor 1 α
IC_50_: the half-maximal inhibitory concentration
ICIs: immune checkpoint inhibitors
JAK: Janus kinase
JNK: c-Jun N-terminal kinase
KD: kinase domain
Keap1/Nrf2: Kelch-like ECH-associated protein 1/nuclear factor erythroid 2-related factor 2
MAPK: mitogen-activated protein kinase
MCL-1: C-Myeloid cell leukemia 1
MeSH: Medical Subject Headings
MIC: minimum inhibitory concentration
miRNAs: microRNAs
MMP: mitochondrial membrane potential
MMP2: matrix metalloproteinase-2
MOMP: mitochondrial membrane permeability
mTOR: mammalian target of rapamycin
mTORC1: mTOR complex 1
MyD88: myeloid differentiation primary response 88
NCCN: National Comprehensive Cancer Network
ncRNA: non-coding RNA
NF-κB: nuclear factor-kappa B
NK: natural killer
OC: ovarian cancer
OS: overall survival
PARP: poly(ADP-ribose) polymerase
PARPi: PARP inhibitors
PDOs: patient-derived organoids
PFS: progression-free survival
PH: pleckstrin homology domain
PI3K: phosphatidylinositol 3-kinase
PIP3: phosphatidylinositol-3,4,5-trisphosphate
PLOOH: phospholipid hydroperoxides
PTEN: phosphorylation and phosphatase and tensin homolog
RECK: reversion-inducing cysteine-rich protein with Kazal motifs
ROS: reactive oxygen species
SAR: structure-activity relationship
SSBs: single-strand breaks
STAT: signal transducer and activator of transcription
STAT3: signal transducer and activator of transcription 3
TIIA: Tanshinone IIA
TAMs: tumor-associated macrophages
TCM: traditional Chinese medicine
TFEB: transcription factor EB
TGF-β: transforming growth factor-β
TIME: tumor immune microenvironment
TLR4: Toll-like receptor 4
TME: tumor microenvironment
TRAIL: TNF-related apoptosis-inducing ligand
Tregs: regulatory T cells
ULK1: unc-51-like kinase 1
VEGF: vascular endothelial growth factor
VEGFR: VEGF receptor
WEE1: WEE1 G2 checkpoint kinase
